# Solubility Thermodynamics of CyMe$_{4}$-BTBP in Various Diluents Mixed with TBP

**DOI:** 10.1007/s10953-018-0774-4

**Published:** 2018-06-25

**Authors:** Jenny Halleröd, Chrisitan Ekberg, Ivan Kajan, Emma Aneheim

**Affiliations:** Nuclear Chemistry, Department of Chemistry and Chemical Engineering, Chalmers University of Technology, Kemivägen 4, 412 58 Gothenburg, Sweden

**Keywords:** CyMe$_{4}$-BTBP solubility, Solvent extraction, Solubility thermodynamics, GANEX process, Used nuclear fuel

## Abstract

The two organic ligands 6,6′-bis(5,5,8,8-tetramethyl-5,6,7,8-tetrahydrobenzo[1,2,4]triazin-3-yl)[2,2′]bipyridine (CyMe$_{4}$-BTBP) and tri-butyl phosphate (TBP) have previously been investigated in different diluents for use within recycling of used nuclear fuel through solvent extraction. The thermodynamic parameters, $K_{\mathrm{S}}$, $\Delta C_{p}$, $\Delta H^{0}$ and $\Delta S^{0}$, of the CyMe$_{4}$-BTBP solubility in three diluents (cyclohexanone, octanol and phenyl trifluoromethyl sulfone) mixed with TBP have been studied at 288, 298 and 308 K, both as pristine solutions and pre-equilibrated with 4 mol$\cdot$L$^{-1}$ nitric acid. In addition, the amount of acid in the organic phase and density change after pre-equilibration have been measured. The solubility of CyMe$_{4}$-BTBP increases with an increased temperature in all systems, especially after acid pre-equilibration. This increased CyMe$_{4}$-BTBP solubility after pre-equilibration could be explained by acid dissolution into the solvent. Comparing the $\Delta H^{0}$ and $\Delta S^{0}$ calculated using $\Delta C_{p}$ with the same parameters derived from a linear fit indicates temperature independence of all three thermodynamic systems. The change in enthalpy is positive in all solutions.

## Introduction

Nuclear power plants, just like other sources of energy, produce significant amounts of secondary waste [1–3]. The most important form of secondary waste from nuclear energy is the used nuclear fuel [1]. This used fuel is highly radiotoxic and has to be isolated from mankind and the environment for a long time [4]. By partitioning and transmutation of the present long-lived actinides, shorter-lived or even stable nuclides can, however, be created instead. The removal of the long-lived actinides will decrease the long-term radiotoxicity of the used fuel, as well as the heat load of the waste, making the final storage more volume-efficient [5–10]. To be able to transmute the actinides they have to be separated from the rest of the used fuel, due to the high neutron capture cross section of some of the lanthanides [11]. Consequently, separation methods where a large fraction of the actinides can be partitioned from the lanthanides within one single step are beneficial.

The separation between trivalent actinides and trivalent lanthanides is difficult due to their similar chemical natures. Solvent extraction processes using bis-triazine-bi-pyridine (BTBP)-type ligand have, however, shown that sufficient separation can be obtained [12–14].

BTBP-type ligands have been suggested for actinide/lanthanide separation in many different types of solvent extraction processes [15–17], one of these being the grouped actinide extraction (GANEX) process [18], originally developed in France [19, 20]. High solubility of the ligand is often desirable, especially in the recycling of transmutation fuel with high minor actinide content [21]. The choice of diluent has previously been shown to influence not only the solubility of the ligands but also, correlated with this, the extraction efficiency of the ligand [18, 21, 22]. In solvent extraction the diluent is also in constant contact with an aqueous phase, which has proven to affect different factors such as extraction system stability towards both ageing and irradiation [23, 24]. The effect of contact of the diluent with acid on ligand solubility has not previously been reported, which is why, in this work, the solubility of CyMe$_{4}$-BTBP in three different solvent compositions (70%$_{\mathrm{vol}}$ cyclohexanone and 30%$_{\mathrm{vol}}$ TBP, 70%$_{\mathrm{vol}}$ 1-octanol and 30%$_{\mathrm{vol}}$ TBP, 70%$_{\mathrm{vol}}$ FS-13 and 30%$_{\mathrm{vol}}$ TBP) has been investigated as a function of acid pre-equilibration of the solvent and of temperature. Thermodynamic data has also been retrieved in order to explain the solvent behavior.

### Background

**Fig. 1:**
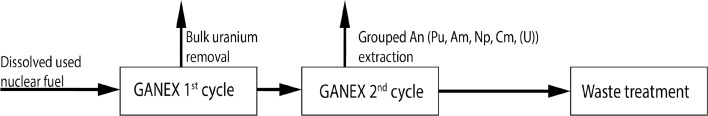
Concept of the GANEX process

The basic concept of the GANEX process is a separation system where the actinides should be extracted together as a group from the fission products, including the lanthanides, and corrosion/activation products. Afterwards the actinides are stripped for transmutation purposes, as illustrated in Fig. 1.

**Fig. 2:**
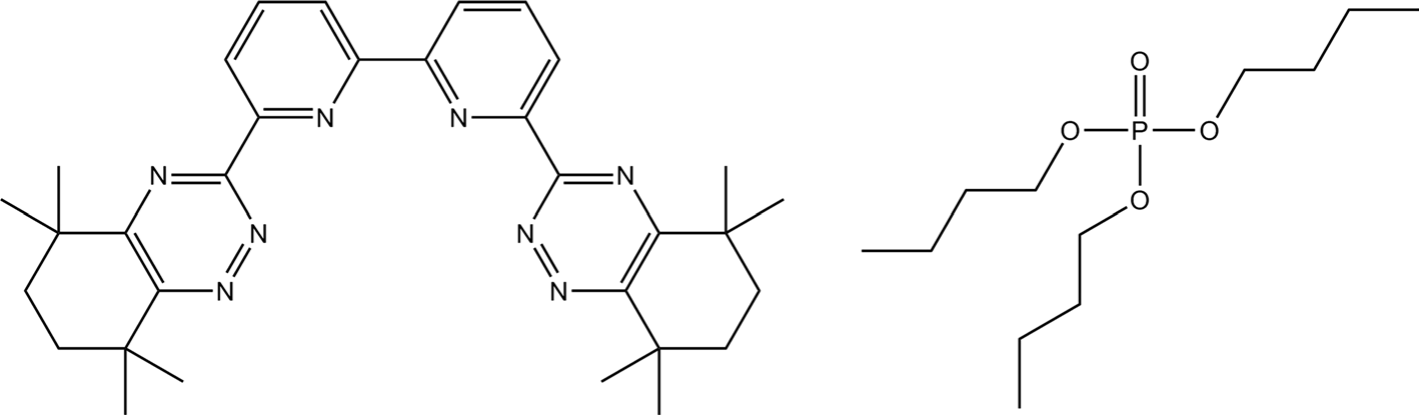
Molecular structure: left: 6,6′-bis(5,5,8,8-tetramethyl-5,6,7,8-tetrahydrobenzo[1,2,4]triazin-3-yl)[2,2′]bipyridine (CyMe$_{4}$-BTBP) and right: tri-butyl phosphate (TBP)

The Chalmers GANEX process is based on the principle of combining two well-known extraction agents in one diluent, making it possible to utilize their separate specific properties. One of the ligands, 6,6′-bis(5,5,8,8-tetramethyl-5,6,7,8-tetrahydrobenzo[1,2,4]triazin-3-yl)[2,2′]bipyridine, CyMe$_{4}$-BTBP, extracts mostly tri- and pentavalent actinides and the other ligand, tri-butyl phosphate, TBP, extracts mainly tetra- and hexavalent actinides. The combination of these two ligands, shown in Fig. 2, has been shown in several papers to be successful for achieving a GANEX separation [18, 25]. The extraction thermodynamics of CyMe$_{4}$-BTBP has previously been investigated in various diluents [26].

### Diluents

Three different diluents have been investigated in this study, phenyl trifluoromethyl sulfone (FS-13), cyclohexanone and 1-octanol. Several different characteristics are desirable in a diluent for use within partitioning and transmutation, such as high solubility of the extracting agents, high flashpoint, stability under highly acidic conditions, low water solubility, stability against radiation and resilience towards third phase formation [27]. In previous studies a correlation between the high CyMe$_{4}$-BTBP solubility and the polarity of the diluent has been found. This indicates that a high polarity of the organic diluent is a prerequisite for good solubility of CyMe$_{4}$-BTBP [21, 28, 29]. A high solubility of CyMe$_{4}$-BTBP increases the possibility of reaching a higher distribution ratio of trivalent actinides, which are not extracted by TBP. This is of particular interest during actinide loading or during recycling of transmuted nuclear fuel where the minor actinide content is high [30].

Cyclohexanone is a diluent that renders a GANEX system with a high distribution ratio of the actinides [18] together with a high solubility of CyMe$_{4}$-BTBP in pure cyclohexanone [21]. Cyclohexanone has drawbacks, however, such as a low flashpoint (Table 1), solubility in the acidic aqueous phase [31] and exothermic reactions with concentrated nitric acid, forming adipic acid [32]. These negative features mean that cyclohexanone is not an ideal diluent for a GANEX process, although similar extraction selectivity and CyMe$_{4}$-BTBP solubility is desirable in an alternative diluent.

1-octanol is a long chained alcohol that has been used in other actinide schemes developed in Europe [15, 33]. It has a lower CyMe$_{4}$-BTBP solubility than cyclohexanone [21] but high distribution ratios for the actinides, and a good extraction selectivity can hence still be achieved [34]. The flashpoint is also higher than that of cyclohexanone (Table 1) [35].

FS-13 was initially developed for the UNiversal EXtraction (UNEX) process and was found to be stable towards nitric acid and resistant against radiation [36, 37]. FS-13 has a high density, a low viscosity [38] and is a polar diluent with good chemical stability [39]. Chalmers GANEX systems using FS-13 as a diluent display sufficient stability against radiation, a high actinide extraction [23] and a selective extraction of the different metals [40].

**Table 1:** Properties of cyclohexanone, 1-octanol, FS-13 and TBP from literature

	Cyclohexanone	1-Octanol
Flash point	317 K^a^	358 K^a^
Water in solvent [wt%]	8.0^b^	4.2^c^
Solvent in water [wt%]	2.3^b^	0.0538^b^
Structure		

	FS-13	TBP
Flash point	395 K^d^	419 KC^e^
Water in solvent [wt%]	–	4.67^b^
Solvent in water [wt%]	–	0.039^b^
Structure		Fig. 2 right

Data marked: ^a^has been retrieved from Ref. [41], ^b^from Ref. [42], ^c^from Ref. [43], ^d^from Ref. [44], ^e^from Ref. [45] and '–' denotes no literature data available. Solubility data retrieved at 298 K

## Theory

### Dissolution Thermodynamics

The dissolution of an organic solid, such as a BTBP-type molecule, in a diluent can be expressed as an equilibrium between the substance in its solid and dissolved forms:

1 $$\begin{aligned} \mathrm{BTBP(s)} \rightleftharpoons \mathrm{BTBP(org)} \end{aligned}$$

The thermodynamic solubility constant *K*$^{0}$ is described as an equilibrium expression between the activity of the solid and dissolved compound. As the activity of a solid is defined to be unity, *K*$^{0}$ is equal to the activity of the dissolved compound (Eq. 2).

2 $$\begin{aligned} K^{0} = \lbrace \mathrm{BTBP(org)}\rbrace / \lbrace \mathrm{BTBP(s)}\rbrace = \lbrace \mathrm{BTBP(org)}\rbrace / 1 \end{aligned}$$

The Gibbs energy, *G*, can be described in several different ways. During dissolution it can be described according to Eq. 3.

3 $$\begin{aligned} \Delta G^{0} = - R T \mathrm{ln} K^{0} = \Delta H^{0} - T \Delta S^{0} \end{aligned}$$

where *R* is the gas constant, *H* is the enthalpy of dissolution and *S* is the entropy of dissolution.

The activity of a substance in solution can be described in terms of concentration by multiplying the concentration with an activity coefficient, $\gamma$. The solubility constant $K_{\mathrm{S}}$ can then be expressed as $K^{0}$ divided by $\gamma$, rendering $K_{\mathrm{S}}$ equal to the concentration of the dissolved compound.

Combining these two equations for the Gibbs energy, enthalpy and entropy of the solubility reactions can be estimated based on the solubility constants obtained from the measurements. There are two different ways to calculate the enthalpy and entropy of a reaction. The van’t Hoff equation, Eq. 4, can be used to obtain a linear extrapolation, where $-\Delta H/R$ represents the slope and $\Delta S/R$ represents the intercept of the linear fit.

4 $$\begin{aligned} \mathrm{ln} K_{\mathrm{S}} = - \Delta H^{0} / (R T) + \Delta S^{0} / R \end{aligned}$$

The van’t Hoff equation is, however, based on the assumption that the enthalpy and entropy are constant with temperature change. In the case of a temperature dependence, the entropy and enthalpy can be calculated through the use of heat capacity and the standard Gibbs energy for each reaction, Eq. 5.

5 $$\begin{aligned} \Delta G^{0}_{T_{\mathrm{X}}} = \Delta G^{0}_{T_{\mathrm{Z}}} - \Delta S^{0}_{T_{\mathrm{Z}}}(T_{\mathrm{X}} - T_{\mathrm{Z}}) - T_{\mathrm{X}} \int _{T_{\mathrm{Z}}}^{T_{\mathrm{X}}} (C_{p}/T) \ dT \ + \ \int _{T_{\mathrm{Z}}}^{T_{\mathrm{X}}} C_{p} \ d T \end{aligned}$$

where $\Delta C_{p}$ is the heat capacity.

By combining Eq. 5 with Eq. 3, the following expressions can be derived, Eqs. 6–8.

6 $$\begin{aligned} \Delta H^{0}_{T_{2}} + \Delta C_{p} ((T_{1}-T_{2}) - T_{1} \mathrm{ln}(T_{1} / T_{2})) - T_{1} \Delta S_{T_{2}}^{0} \nonumber = - R \Delta T_{1} \mathrm{ln}\ K_{\mathrm{S}_{1}} \end{aligned}$$

7 $$\begin{aligned} \Delta H^{0}_{T_{2}} - T_{2} \Delta S_{T_{2}}^{0}= & {} - R T_{2} \mathrm{ln} K_{\mathrm{S}_{2}}\end{aligned}$$

8 $$\begin{aligned} \Delta H^{0}_{T_{2}} + \Delta C_{p} ((T_{3}-T_{2}) - T_{3} \mathrm{ln}(T_{3} / T_{2})) - T_{3} \Delta S_{T_{2}}^{0} \nonumber = - R \Delta T_{3} {\mathrm{ln}} K_{\mathrm{S}_{3}} \end{aligned}$$

The solution can be considered temperature independent within the studied temperature interval, if the obtained enthalpy and entropy from the linear fits match the calculated enthalpy and entropy.

## Experimental

### Solubility

The solubility of CyMe$_{4}$-BTBP was investigated in three different GANEX solvents based on the diluents FS-13 (Marshallton, $\ge$ 99%), cyclohexanone (Sigma–Aldrich, $\ge$ 99.0%) and 1-octanol (Sigma–Aldrich, $\ge$ 99.0%). Each solution was prepared with 70%$_{\mathrm{vol}}$ diluent and 30%$_{\mathrm{vol}}$ TBP (Sigma–Aldrich, 97%) in two different batches, where one was pristine and the other pre-equilibrated with 4 mol$\cdot$L$^{-1}$ nitric acid, Table 2.

**Table 2:** Organic phase content in the different solutions

Solution	Content
FS-13/TBP^a^	70%$_{\mathrm{vol}}$ FS-13, 30%$_{\mathrm{vol}}$ TBP
FS-13/TBP^b^	70%$_{\mathrm{vol}}$ FS-13, 30%$_{\mathrm{vol}}$ TBP pre-equilibrated with HNO$_{3}$
cyclohex./TBP^a^	70%$_{\mathrm{vol}}$ cyclohexanone, 30%$_{\mathrm{vol}}$ TBP
cyclohex./TBP^b^	70%$_{\mathrm{vol}}$ cyclohexanone, 30%$_{\mathrm{vol}}$ TBP pre-equilibrated with HNO$_{3}$
octanol/TBP^a^	70%$_{\mathrm{vol}}$ 1-octanol, 30%$_{\mathrm{vol}}$ TBP
octanol/TBP^b^	70%$_{\mathrm{vol}}$ 1-octanol, 30%$_{\mathrm{vol}}$ TBP pre-equilibrated with HNO$_{3}$

All samples were prepared at 294 K and atmospheric pressure 0.1 MPa

Standard uncertainties, u, are u(*T*) = ± 0.01 K and u(*p*) = ± 0.01 MPa

The 4 mol$$\cdot $$L$$^{-1}$$ HNO$$_{3}$$ was prepared from concentrated HNO$$_{3}$$ (Sigma–Aldrich, $$\ge $$ 69%) diluted in Milli-Q water (Milipore, > 18 M$$\Omega $$$$\cdot $$cm^−1^). In the pre-equilibrated solutions the organic phase and nitric acid were thoroughly mixed and centrifuged at room temperature (294 K) before the organic phases were separated from the acid prior to CyMe$$_{4}$$-BTBP dissolution.

1 mL of each solution was placed in a glass vial together with a large amount of solid CyMe$_{4}$-BTBP (synthesized in-house according to [46] and at the University of Reading). The samples were placed in a thermostated mechanical shaker (IKA, VIBRAX VXR 1500 rpm) at the desired temperature and left for three days. During these three days additional CyMe$_{4}$-BTBP was added morning and evening until undissolved CyMe$_{4}$-BTBP was observed. The samples were maintained at the same temperature as the experiment for two additional days to allow the solid phase to settle. Each experiment was performed in triplicate and at three different temperatures: 288, 298 and 308 K.

### Solubility Analysis

The quantifications of CyMe$_{4}$-BTBP solubility were based on the ability of CyMe$_{4}$-BTBP to form a blue-colored complex with Fe$^{2+}$ ions in solution [21]. The absorbance of the blue complex between CyMe$_{4}$-BTBP and Fe$^{2+}$ was measured using quartz cuvettes with a 10 mm path length at a wavelength of 598 nm using a UV–VIS spectrophotometer (Shimadzu UV-1800). A solution of Mohrs salt (Sigma–Aldrich, p.a. 99%) was used as the source of Fe$^{2+}$ ions. This solution was prepared using an excess of solid Mohrs salt dissolved in 50 mL ethanol (Solveco, 95%) and 50 mL Milli-Q water, added in the order stated. A fresh iron solution was prepared prior to each measurement, since the solution is sensitive towards ageing as Fe$^{2+}$ is oxidized to Fe$^{3+}$.

For the spectrophotometry measurement 0.1–0.3 mL of the solvent with dissolved CyMe$_{4}$-BTBP was added to a solution containing 10 mL of ethanol and 0.5 mL Fe$^{2+}$ solution. A mixture of 70%$_{\mathrm{vol}}$ diluent and 30%$_{\mathrm{vol}}$ TBP was then added to make up a total volume of 0.4 mL of added organic phase. Absorption was measured against a reference solution containing 0.4 mL solvent without CyMe$_{4}$-BTBP in the 10 mL of ethanol and 0.5 mL Fe$^{2+}$ solution. Solutions with known concentrations of CyMe$_{4}$-BTBP were used to obtain a calibration curve prior to the measurements. The different diluents had no visible effect on the shape of the calibration curve and the absorption spectra.

### Density, Surface Tension and Acid Extraction

The density, $\rho$, and surface tension, $\gamma$, against air (using the du Noüy ring method [47]) for the different solvents was measured at room temperature (293–295 K) using a tensiometer (Sigma 700, Attension). The surface tensiometer ring was lowered into the organic phase until it was totally immersed. The tensiometer then measured the surface tension automatically by slowly pulling out the ring of the organic phase and measuring the force required to detach the ring from the organic surface. The density was measured using the same tensiometer and a calibrated density probe.

Possible acid extraction/protonation of the organic phase upon pre-equilibration was investigated using 4 mol$\cdot$L$^{-1}$ nitric acid spiked with tritium. Equal parts of tritium-spiked nitric acid and organic phase were mixed and left to separate. Samples were taken from each phase and measured in a liquid scintillation counting detector (Wallac 1414 WinSpectral).

## Results and Discussion

**Fig. 3:**
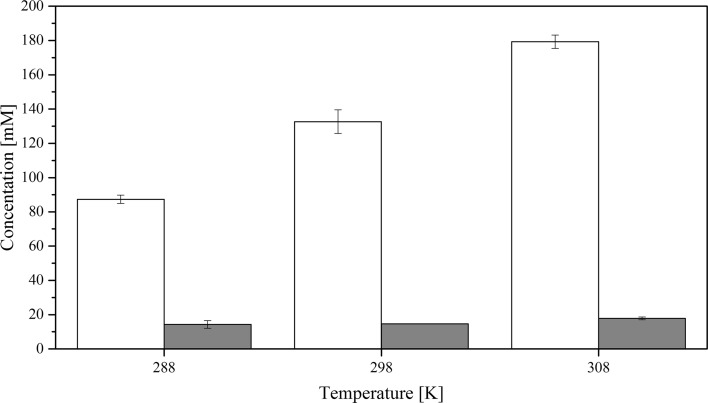
Solubility of CyMe$_{4}$-BTBP in 70%$_{\mathrm{vol}}$ FS-13 and 30%$_{\mathrm{vol}}$ TBP where mM denotes the concentration in mmol$\cdot$L^−1^; the white bars show the pre-equilibrated organic phase and the grey bars show the pristine organic phase. The uncertainties are standard deviations

**Fig. 4:**
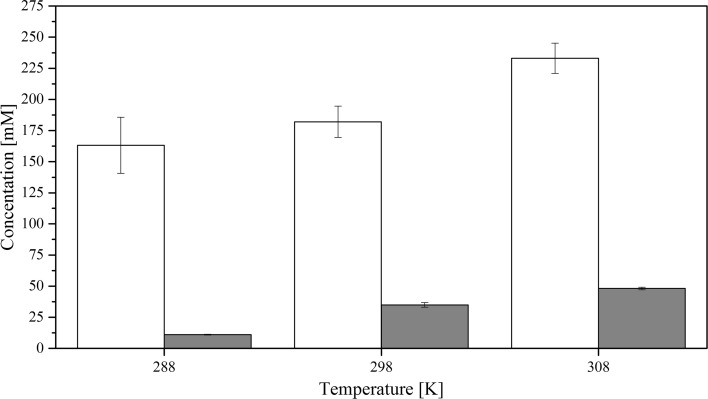
Solubility of CyMe$_{4}$-BTBP in 70%$_{\mathrm{vol}}$ cyclohexanone and 30%$_{\mathrm{vol}}$ TBP where mM denotes the concentration in mmol$\cdot$L^−1^; the white bars show pre-equilibrated organic phase and the grey bars show pristine organic phase. The uncertainties are standard deviations

**Fig. 5:**
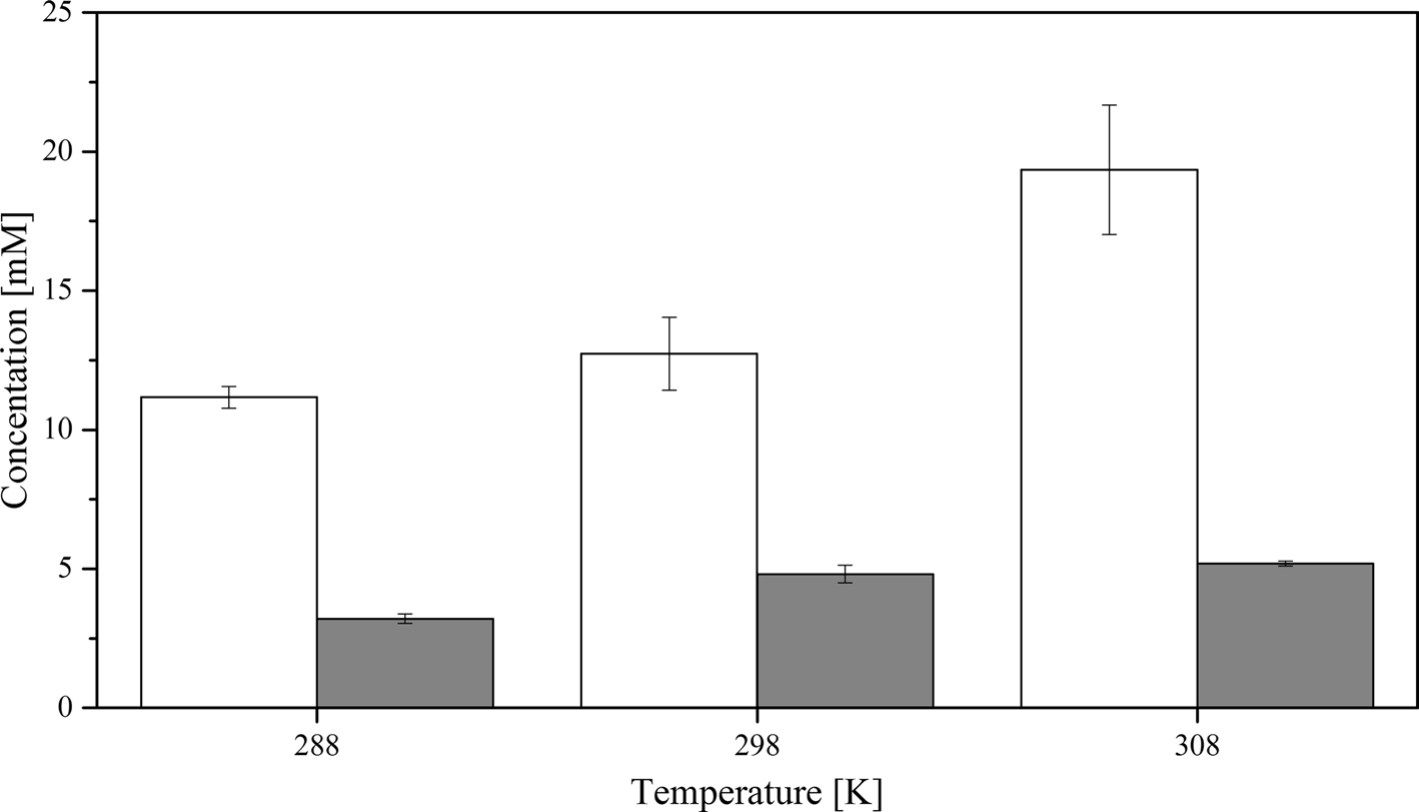
Solubility of CyMe$_{4}$-BTBP in 70%$_{\mathrm{vol}}$ 1-octanol and 30%$_{\mathrm{vol}}$ TBP where mM denotes the concentration in mmol$\cdot$L^−1^; the white bars show pre-equilibrated organic phase and the grey bars show pristine organic phase. The uncertainties are standard deviations

Experiments show that the overall solubility of CyMe$_{4}$-BTBP in FS-13, cyclohexanone and 1-octanol-based organic phases containing 30%$_{\mathrm{vol}}$ TBP increase drastically in all solvents when the organic phases are pre-equilibrated with 4 mol$\cdot$L$^{-1}$ HNO$_{3}$ compared to the pure solvent, see Figs. 3, 4 and 5. No aqueous phase was present during the dissolution. Contrary to previous studies [21], the high solubility of ligand, especially in the FS-13/TBP diluent combination, does not correspond to an increased actinide extraction efficiency at a given temperature, Table 3.

**Table 3:** Americium distribution ratio and CyMe$_{4}$-BTBP solubility in investigated solutions

Solvent composition	CyMe$_{4}$-BTBP solubility at 298 K	Americium distribution ratio at 298 K using 10 mmol$\cdot$L$^{-1}$ CyMe$_{4}$-BTBP
FS-13/TBP	130	30^a^
Cyclohex./TBP	180	160^b^
Octanol/TBP	13	30^c^

70%$_{\mathrm{vol}}$ diluent and 30%$_{\mathrm{vol}}$ TBP in all cases. 10 mmol$\cdot$L$^{-1}$ CyMe$_{4}$-BTBP was used for extraction. Data marked: ^a^has been retrieved from Ref. [48], ^b^from Ref. [49] and ^c^from Ref. [34]

The results in Figs. 3, 4 and 5 also indicate that the solubility of CyMe$_{4}$-BTBP increases with increased temperature, both for the pristine organic phase and the pre-equilibrated phase. The difference in solubility between the pristine and the pre-equilibrated solvents also increases with increasing temperature. The higher solubility of CyMe$_{4}$-BTBP in the pre-equilibrated organic phase indicates that the aqueous phase plays a significant role in the solubility by affecting the organic phase. It has previously been shown that CyMe$_{4}$-BTBP is not soluble above the detection limit in a nitric acid aqueous phase [50]. It is known that HNO$_{3}$ is extracted to a larger degree than HCl [51, 52] while having a similar acid dissociation; single samples with 70%$_{\mathrm{vol}}$ FS-13 and 30%$_{\mathrm{vol}}$ TBP where the 4 mol$\cdot$L$^{-1}$ nitric acid was exchanged with 4 mol$\cdot$L$^{-1}$ hydrochloric acid (with acid dissociation of 3.9 and 4.0 respectively), Milli-Q water and 4 mol$\cdot$L$^{-1}$ NaCl dissolved in Milli-Q water, to rule out the effect of chloride, were prepared to obtain an indication of whether the increased solubility is a result of nitric acid extraction as non-dissociated HNO$_{3}$, protonation of the organic phase or another phenomenon. The sample containing HCl reached a CyMe$_{4}$-BTBP solubility of 30 $\times$ 10$^{-3}$ mol$\cdot$L$^{-1}$, the sample containing Milli-Q water reached a CyMe$_{4}$-BTBP solubility of 15 $\times$ 10$^{-3}$ mol$\cdot$L$^{-1}$ and the sample containing NaCl reached a CyMe$_{4}$-BTBP solubility of 20 $\times$ 10$^{-3}$ mol$\cdot$L$^{-1}$. These results, compared to the CyMe$_{4}$-BTBP solubility of 130 $\times$ 10$^{-3}$ mol$\cdot$L$^{-1}$ using HNO$_{3}$, are of the same magnitude, indicating that it is the nitrate or the extraction of undissociated HNO$_{3}$ rather than the acidity or protonation that is crucial for the increased CyMe$_{4}$-BTBP solubility.

Proton and chloride distribution between the FS-13/TBP organic phase (without CyMe$_{4}$-BTBP) and different aqueous phases was investigated using an aqueous phase spiked with tritium or Cl-36, by measuring the activity in the two phases. Besides investigating 70%$_{\mathrm{vol}}$ FS-13 and 30%$_{\mathrm{vol}}$ TBP and 4 mol$\cdot$L$^{-1}$ nitric acid spiked with tritium, three single samples with 70%$_{\mathrm{vol}}$ FS-13 and 30%$_{\mathrm{vol}}$ TBP were prepared where the 4 mol$\cdot$L$^{-1}$ nitric acid was exchanged with Milli-Q water spiked with tritium, 4 mol$\cdot$L$^{-1}$ hydrochloric acid spiked with tritium and 4 mol$\cdot$L$^{-1}$ hydrochloric acid spiked with Cl-36. In the sample containing Milli-Q water 20% of the tritium was extracted by the organic phase, while 28% of the tritium was extracted by the sample containing hydrochloric acid compared to 15% using nitric acid. Only 1.8% of the Cl-36 was extracted by the organic phase. This indicates that the protons detected in the organic phase mainly originate from dissolved water and that, as could be expected, HCl is only extracted in small amounts.

In addition, the change in density and surface tension of the solvents with pre-equilibration was also measured. According to the results presented in Table 4, the surface tensions of the solvents are not influenced by pre-equilibration. However, a large amount of tritium originating from the aqueous phase was found in all three pre-equilibrated organic phases, as well as a slight increase in density. The measured densities correspond well with the theoretical densities of the solvent based on a linear combination of the separate densities of the diluents and TBP. Literature data for the density of 1-octanol mixed with TBP [53] and for FS-13 [54] correspond well with the measured data.

**Table 4:** Surface tensions, $\gamma$, densities, $\rho$, and amounts tritium, $^{3}$H, in the organic phase of the different solutions

Solution	Surface tension (mN$\cdot$m$^{-1}$)	Density (g$\cdot$cm$^{-3}$)	% $^{3}$H in organic phase
FS-13	26.6 ± 0.01	1.40 ± 0.01	
FS-13/TBP a	28.5 ± 0.01	1.26 ± 0.001	
FS-13/TBP b	28.9 ± 0.01	1.28 ± 0.003	15% ± 0.4
Ideal values FS-13/TBP		1.27	
Cyclohexanone	34.4^a^	0.95^b^	
Cyclohex./TBP a	31.2 ± 0.02	0.95 ± 0.003	
Cyclohex./TBP b	31.3 ± 0.01	0.98 ± 0.01	21% ± 0.7
Ideal values Cyclohex./TBP		0.96	
1-Octanol	27.2^a^	0.83^b^	
Octanol/TBP a	27.2 ± 0.01	0.88 ± 0.02	
Octanol/TBP b	27.2 ± 0.01	0.89 ± 0.002	25% ± 0.4
Ideal values Octanol/TBP		0.87	
TBP	27.6^c^	0.97^b^	
4 mol$\cdot$L$^{-1}$ HNO$_{3}$		1.14 ± 0.01	

In all solutions containing a mixture of TBP and diluent, 30%$_{\mathrm{vol}}$ TBP and 70%$_{\mathrm{vol}}$ diluent was used and 'a' represents the pristine organic phase and 'b' represents the pre-equilibrated organic phase. Ideal values are based on a linear combination of 70%$_{\mathrm{vol}}$ diluent and 30%$_{\mathrm{vol}}$ TBP. The uncertainties are standard deviations. Surface tension and density data were collected at a temperature of 294 K, the tritium data wrere collected at *T* = (288, 298 and 308) K and found to be constant. All data were collected at an atmospheric pressure of 0.1 MPa

Data marked: ^a^has been retrieved from Ref. [55], ^b^from Ref. [41] and ^c^from Ref. [45].

Standard uncertainties, u, are u(*T*) = ± 0.01 K and u(*p*) = ± 0.01 MPa.

The statistical uncertainties are with a 95% confidence interval

The presence of tritium in the organic phase and the slight increase in density strengthen the claim that the aqueous phase affects the organic phase upon pre-equilibration. It has previously been found that TBP extracts nitric acid as a complex [56], which can change the properties of the organic phase. Other possibilities are protonation or acid extraction of the diluent, which might change the polarity of the system and change the solubility, or in turn cause protonation of CyMe$_{4}$-BTBP. Both cyclohexanone [57] and 1-octanol [58] are known to extract nitric acid.

**Fig. 6:**
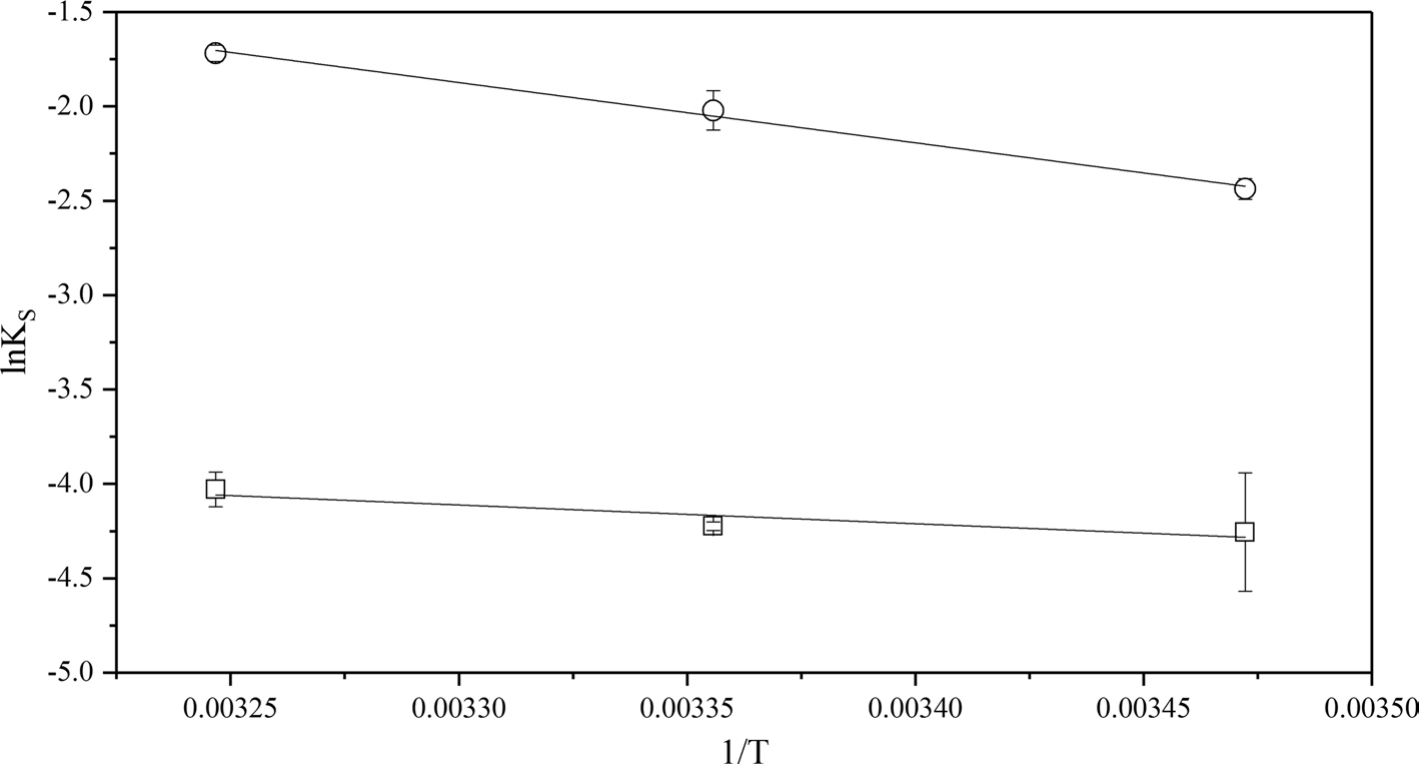
The dependence of the natural logarithm of CyMe$_{4}$-BTBP solubility in 70%$_{\mathrm{vol}}$ FS-13 and 30%$_{\mathrm{vol}}$ TBP, circles ($\circ$) with pre-equilibrated organic phase and rectangles ($\Box$) with pristine organic phase, plotted versus 1/*T*

**Fig. 7:**
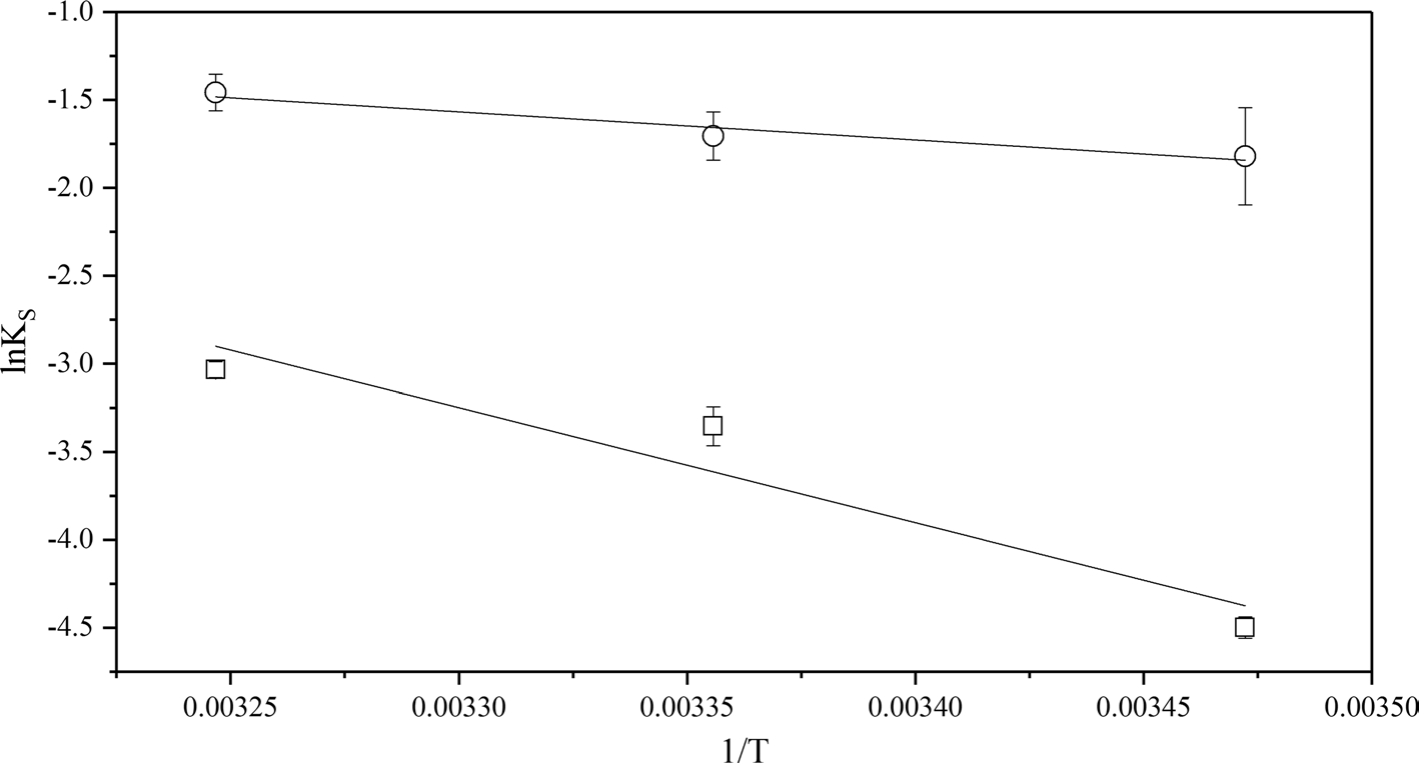
The dependence of the natural logarithm of CyMe$_{4}$-BTBP solubility in 70%$_{\mathrm{vol}}$ cyclohexanone and 30%$_{\mathrm{vol}}$ TBP, circles ($\circ$) with pre-equilibrated organic phase and rectangles ($\Box$) with pristine organic phase, plotted versus 1/*T*

**Fig. 8:**
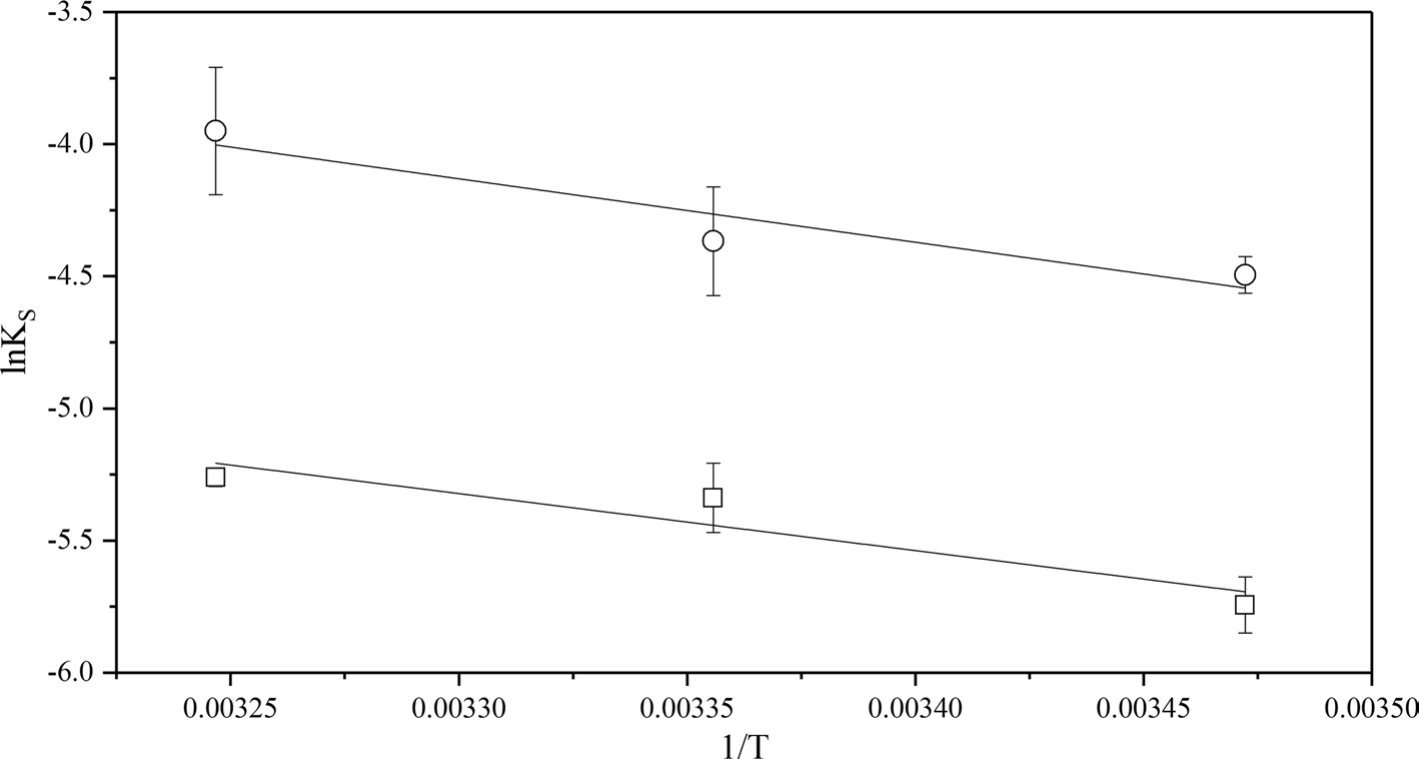
The dependence of the natural logarithm of CyMe$_{4}$-BTBP solubility in 70%$_{\mathrm{vol}}$ 1-octanol and 30%$_{\mathrm{vol}}$ TBP, circles ($\circ$) with pre-equilibrated organic phase and rectangles ($\Box$) with pristine organic phase, plotted versus 1/*T*

The natural logarithm of the solubility constant, Eq. 4, at different temperatures was plotted against 1/*T*, see Figs. 6, 7 and 8. The solubility constant of CyMe$_{4}$-BTBP increases in all three diluents with increasing temperature, indicating that the solubility reaction is endothermic.

The values of enthalpy, $\Delta H^{0}$, and entropy, $\Delta S^{0}$, were calculated for both the pristine and the acid pre-equilibrated systems. The values of ln$K_{\mathrm{S}}$ for the pristine solvents in Figs. 7 and 8 deviates slightly from linearity, indicating a non-linear temperature effect on the CyMe$_{4}$-BTBP solubility. This could be explained by a non-uniform change in the heat transfer or by errors in the measurements. Therefore, the linear fit results obtained from Eq. 4 using data from Figs. 6, 7 and 8 were also compared with results obtained from calculations using the heat capacity, $\Delta C_{p}$, according to Eqs. 6–8, see Table 5.

**Table 5:** Enthalpy, $\Delta H^{0}$, entropy $\Delta S^{0}$ and heat capacity, $\Delta C_{p}$, changes obtained for the solvents used

Solution	$\Delta H^{0}$ (kJ$\cdot$mol$^{-1}$)	$\Delta S^{0}$ (J$\cdot$mol$^{-1}\cdot$K$^{-1}$)	$\Delta C_{p}$ (kJ$\cdot$mol$^{-1}\cdot$ K$^{-1}$)	$\Delta H^{0}$ linear (kJ$\cdot$mol$^{-1}$)	$\Delta S^{0}$ linear (J$\cdot$mol$^{-1}\cdot$ K$^{-1}$)
FS-13/TBP^a^	8	− 6	1.3	8.3 ± 0.6	− 6.9 ± 0.4
FS-13/TBP^b^	27	72	− 0.7	26.6 ± 0.3	72.1 ± 0.2
Cyclohex./TBP^a^	53	150	− 5.7	54.4 ± 2.6	152.4 ± 1.9
Cyclohex./TBP^b^	15	38	1.4	13.3 ± 0.5	30.8 ± 0.3
Octanol/TBP^a^	17	14	− 2.3	18.0 ± 1.1	15.0 ± 0.8
Octanol/TBP^b^	21	33	2.3	20.0 ± 1.0	31.6 ± 0.7

The uncertainties in $\Delta H^{0}$ linear and $\Delta S^{0}$ linear are calculated from the linear regression and the 95% confidence interval of the fitting. The organic phase in the solutions constitutes 70%$_{\mathrm{vol}}$ diluent and 30%$_{\mathrm{vol}}$ TBP. Solution ‘a’ represents the pristine organic phases while solution ‘b’ represents the pre-equilibrated organic phase. All data were collected at *T* = (288, 298 and 308) K and atmospheric pressure of 0.1 MPa. Standard uncertainties, u, are u(*T*) = ± 0.01 K and u(*p*) = ± 0.01 MPa.

The statistical uncertainties are with a 95% confidence interval

It is important to note that the values obtained for $\Delta C_{p}$ are estimated using a very small temperature interval and only three temperatures. This means that the solution is exact without any degrees of freedom left for uncertainty estimations. The results will thus be strongly dependent on any uncertainty in the experimental points. However, since the enthalpies and entropies are comparable the approach still has merit.

When comparing the results for the calculated $\Delta H^{0}$ and $\Delta S^{0}$ using $\Delta C_{p}$ with $\Delta H^{0}$ linear and $\Delta S^{0}$ linear obtained from the linear fit, only small variations are found, indicating temperature independence for all six solvent systems. The change in enthalpy, $\Delta H^{0}$, is positive for all six systems, indicating an endothermic dissolution process; i.e. the systems consume energy from the outer environment. In the 1-octanol and FS-13 based systems both the enthalpy and the entropy increased with acid pre-equilibration. For the cyclohexanone system, however, both the enthalpy and the entropy decreased with acid pre-equilibration. Low values of the change in enthalpy indicate that the bonds between the CyMe$_{4}$-BTBP molecules and the solution are strong. For two systems, such as solutions 2b and 3b that have similar values of $\Delta S^{0}$, the system with the lower value of enthalpy will have a higher CyMe$_{4}$-BTBP solubility, which correlates with experimental data, see Figs. 4 and 5. Solution 1a is the only system with a negative entropy, indicating a small change or no change in the status quo of the system, i.e. the molecular order of the system is decreasing. The negative $\Delta C_{p}$ values in the cases of solutions 1b, 2a and 3a may indicate that the systems lost vibrational or translational degrees of freedom compared to the corresponding systems, solutions 1a, 2b and 3b. From the enthalpies and entropies, Gibbs energies, $\Delta G^{0}$, can be calculated using Eq. 3. Using $\Delta G^{0}$ the systems can be more easily be compared to each other, since when $\Delta G^{0}$ decreases, the solubility increases. Combining Eqs. 3 and 2, the solubility corresponding to the obtained $\Delta G^{0}$ values can be calculated. Comparing the calculated solubility results with Figs. 3, 4 and 5, no significant differences are found (Table 6).

**Table 6:** Gibbs energy, $\Delta G^{0}$, and CyMe$_{4}$-BTBP concentrations calculated from the enthalpy and entropy obtained from the linear fits

Solution	$\Delta G^{0}$ (288 K) (kJ$\cdot$mol$^{-1}$)	$\Delta G^{0}$ (298 K) (kJ$\cdot$mol$^{-1}$)	$\Delta G^{0}$ (308 K) (kJ$\cdot$mol$^{-1}$)	*c* (288 K) (mmol$\cdot$L$^{-1}$)	*c* (298 K) (mmol$\cdot$L$^{-1}$)	*c* (308 K) (mmol$\cdot$L$^{-1}$)
FS-13/TBP^a^	10.3	10.3	10.4	14	16	17
FS-13/TBP^b^	5.8	5.1	4.4	90	130	180
Cyclohex./TBP^a^	10.5	8.9	7.4	13	27	56
Cyclohex./TBP^b^	4.4	4.1	3.8	160	190	230
Octanol/TBP^a^	13.6	13.4	13.3	3.4	4.3	5.5
Octanol/TBP^b^	10.9	10.6	10.2	11	14	18

The organic phase in the solutions constitutes 70%$_{\mathrm{vol}}$ diluent and 30%$_{\mathrm{vol}}$ TBP. Solution ‘a’ represents the pristine organic phases, while solution ‘b’ represents the pre-equilibrated organic phase

## Conclusions

According to the results obtained, a large increase of the CyMe$_{4}$-BTBP solubility occurs when the organic phase is pre-equilibrated with 4 mol$\cdot$L$^{-1}$ HNO$_{3}$. This increase is also more pronounced at higher temperatures. The increased solubility is most likely an effect of the nitric acid influencing the solvent properties. The possibility of dissolving a large amount of ligand is important for the efficient recycling of used nuclear fuel with high plutonium and/or minor actinide content. All solutions, both pre-equilibrated and pristine, are endothermic systems where the solubility of the CyMe$_{4}$-BTBP increases with increasing temperature. These features are beneficial as a solvent extraction process aimed at recycling used nuclear fuel will take place in a highly acid environment and also under elevated temperatures due to the inherent radiation of the used fuel. An opposite scenario, where solubility decreases with an increased temperature, could cause precipitation of ligand and/or complexed metal which severely would impact the safety of the process. Comparing $\Delta H^{0}$ and $\Delta S^{0}$ values calculated using $\Delta C_{p}$ with the same properties derived from linear fit indicates temperature independence of all six thermodynamic systems. There are, however, no thermodynamic trends found between the systems that explain the higher CyMe$_{4}$-BTBP solubility in the pre-equilibrated solutions.
